# Colonic Transendoscopic Enteral Tubing Is a New Pathway to Microbial Therapy, Colonic Drainage, and Host–Microbiota Interaction Research

**DOI:** 10.3390/jcm12030780

**Published:** 2023-01-18

**Authors:** Weihong Wang, Gaochen Lu, Xia Wu, Quan Wen, Faming Zhang

**Affiliations:** 1 Medical Center for Digestive Diseases, The Second Affiliated Hospital of Nanjing Medical University, 121 Jiang Jia Yuan, Nanjing 210011, China; 2 Key Lab of Holistic Integrative Enterology, The Second Affiliated Hospital of Nanjing Medical University, 121 Jiang Jia Yuan, Nanjing 210011, China; 3 Department of Microbiotherapy, Sir Run Run Hospital, Nanjing Medical University, 109 Longmian Avenue, Nanjing 211166, China

**Keywords:** fecal microbiota transplantation, transendoscopic enteral tube, drainage, host–microbiota interactions, proof-of-concept

## Abstract

The limitation of traditional delivery methods for fecal microbiota transplantation (FMT) gave birth to colonic transendoscopic enteral tubing (TET) to address the requirement of frequent FMTs. Colonic TET as a novel endoscopic intervention has received increasing attention in practice since 2015 in China. Emerging studies from multiple centers indicate that colonic TET is a promising, safe, and practical delivery method for microbial therapy and administering medication with high patient satisfaction. Intriguingly, colonic TET has been used to rescue endoscopy-related perforations by draining colonic air and fluid through the TET tube. Recent research based on collecting ileocecal samples through a TET tube has contributed to demonstrating community dynamics in the intestine, and it is expected to be a novel delivery of proof-of-concept in host–microbiota interactions and pharmacological research. The present article aims to review the concept and techniques of TET and to explore microbial therapy, colonic drainage, and microbial research based on colonic TET.

## 1. Introduction

The relationship between the intestinal microbiome and diseases has been studied and documented through developments in the field of microbiology and metabolomics. The essence of microbial therapy is to reconstruct the patient’s gut microbiota, and fecal microbiota transplantation (FMT) is the most common method. Recently, increasing evidence has demonstrated the therapeutic potential of FMT in many diseases including recurrent *Clostridioides difficile* infection (rCDI) [1], inflammatory bowel disease (IBD) [2,3], refractory irritable bowel syndrome [4], autism [5], diabetes mellitus [6], serious antibiotics-associated diarrhea [7], radiation enteritis [8], non-erosive reflux disease [9], and other microbiota-related diseases.

FMT-related delivery methods are traditionally divided into the upper gut, the mid-gut, and the lower gut [10,11,12]. Oral capsule is a delivery method via the upper gut [13]. The mid-gut routes for FMT include the gastroscopy, nasojejunal tube, percutaneous endoscopic gastro-jejunostomy, and mid-gut transendoscopic enteral tubing [14]. The microbiota suspension can be infused into the lower gut through enema, colonoscopy, distal ileum stoma, colostomy, or colonic transendoscopic tubing (TET) [12]. Colonic TET, a novel delivery pathway, was initially designed for multiple FMT and colon administration [12]. Recently, a systematic review reported that patients who underwent colonic TET had the lowest incidence of delivery-related adverse events (AEs) compared with patients using other delivery routes such as capsule, gastroscopy, colonoscopy, and mid-gut tube, etc. [15]. In addition, Allegretti et al. also stated that TET was a promising approach for FMT due to its considerable improvement in safety [16].

Endoscopic placement of an intestinal decompression tube is a practical technique for the treatment of acute intestinal dilation. However, patients with decompression tubes face many difficulties in conducting their daily tasks. Colonic TET, as a delivery method for microbial therapy, could be used to solve the problem of the development of colonic perforation due to IBD or endoscopic-associated injury to avoid surgery [17]. There is a clinical necessity of using a maintainable colonic tube by combining the use of decompression and medication delivery.

Based on the novel implanting method of colonic TET, which directly connects the deep intestine to the exterior, its application is already beyond microbial therapy. The innovation of colonic TET has been proven to be a new non-invasive method of sampling fecal suspension from the cecum for microbiomics and metabolomics research [18]. In addition, colonic TET could represent a novel delivery method for proof-of-concept in pharmacological research. Considering the multiple applications of colonic TET, this review aims to present up-to-date evidence of colonic TET in microbial therapy, colonic drainage, and host–microbiota interaction.

## 2. The Concept and Technique of Colonic TET

**Figure 1:**
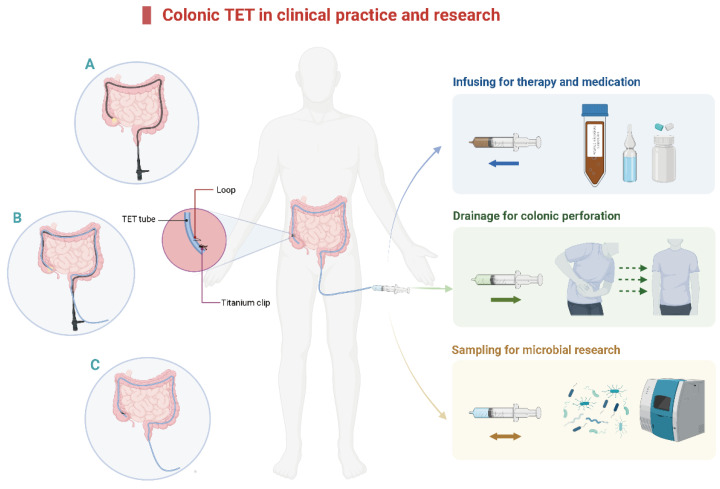
The diagram of colonic TET in clinical practice and research. (**A**). Insert the colonic TET into the endoscopic channel when the endoscope reaches the target location, then remove the endoscope. (**B**). Re-insert the endoscope and hold the TET tube in place. (**C**). Insert the titanium clip to fix the loop of the colonic TET tube onto the intestinal wall. TET: transendoscopic enteral tubing.

The concept of TET was first reported in 2015 [12]. The main clinical applications of colonic TET are shown in Figure 1. A tiny and soft TET tube should be inserted into the deep colon with endoscopic guidance. After that, the endoscope is removed from the colon while the TET tube is maintained at the target location. Then, the endoscope is re-inserted to fix the TET tube in place [12,19]. The colonic TET tube (FMT medical, Nanjing, China) has three separate loops attached to the tube: the first, second, and third site/station. The first loop is fixed to the proximal end of the colon, 10 cm away from the subsequent loop [19]. Each line-loop on the tube is used to fix the tube to the intestinal wall with one or two endoscopic clips (e.g., ROOC-D-26-195-C, ≥10 mm, Nanjing Microtech Co.; HX-610-135 L, 135°, Olympus). The location and number of clips are based on the mucosal folds, disease severity, and duration for which the tube needs to be retained. Generally, 1–2 clips at the first site and 0–2 clips at the second and/or third site (as required) are recommended [12,19,20]. A previous study indicated that using more than four clips had no extra benefit in prolonging the maintenance time of the TET tube [19]. There is a guide wire within the tube for colonic TET, which is removed from the distal TET tube when the colonoscope is withdrawn from the intestine. Subsequently, the TET tube outside the anus is fixed to the hip lubricated with paraffin.

After the endoscope arrives at the target location (e.g., the cecum, ascending colon, transverse colon, and descending colon), the TET tube lubricated with paraffin oil is inserted through the endoscopic channel (diameter >3.2 mm is recommended). A recent study demonstrated that the implantation of a colonic TET tube was quick and safe, although it required double cecal intubation. Compared with regular colonoscopy, the whole cecal intubation time was decreased with the help of cap-assisted colonoscopy, especially the second cecal intubation time (2.8 min vs. 2.2 min, *p* < 0.001) [21].

## 3. The Applications of Colonic TET

### 3.1. Colonic TET for Microbial Therapy

FMT via colonoscopy is impractical for patients who require multiple FMTs to undergo repeated colonoscopy in a short period of time. As for pediatric or older patients who cannot care for themselves, there is a high risk of choking into the airway accidentally when swallowing the oral capsules. Furthermore, it is also not recommended for patients who are unconscious. The microbiota suspension delivered by enema can only reach the rectal and sigmoid colon, making it difficult for patients to retain it in the gut for sufficient time. Therefore, colonic TET was developed to meet the needs of multiple FMTs. In addition, whole or local colonic administration of medications is possible through colonic TET including mesalazine and corticosteroids [12,19]. A study by Oancea et al. demonstrated that the local administration of thioguanine in the rectum might be an effective treatment for colitis [22]. They highlighted the advantages of local drug administration, which reduces the risk of serious side effects associated with systemic delivery. Moreover, colonic TET works for patients who can endure regular enemas. The differences among the common delivery methods are shown in Table 1. The improved methodology of FMT was termed “washed microbiota transplantation” (WMT), which is based on an automatic filtration and washing process and the related delivery [23]. During the washing process, more types, quantities of viruses, and pro-inflammatory mediators are washed out to improve the safety of WMT. Recently, Lu et al. found that colonic TET for delivering WMT was the predominant method used in ulcerative colitis (UC, 67.2%) [24].

**Figure 2:**
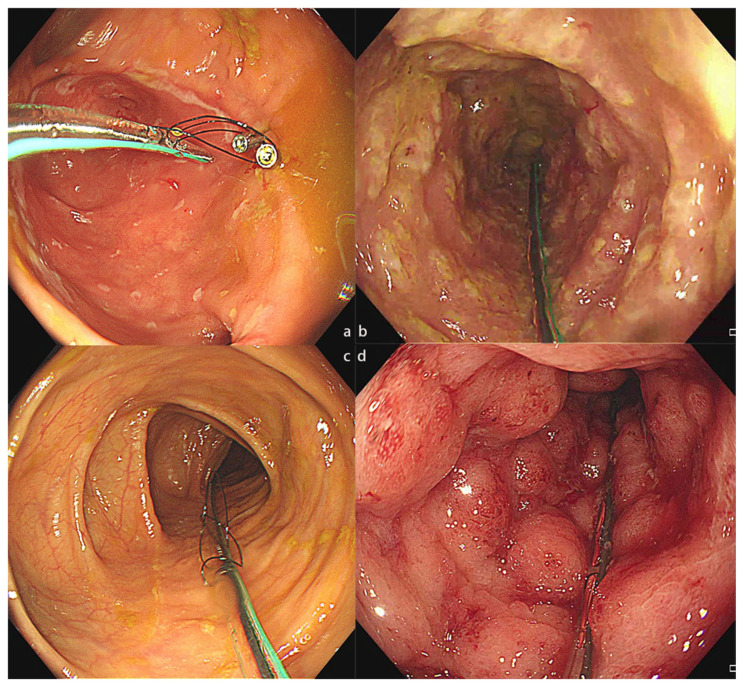
The colonic TET tube used in different disease conditions. (**a**) The colonic TET tube in the intestine of a patient with CD. (**b**). The colonic TET tube in the intestine of a patient with rCDI. (**c**) The colonic TET tube in the intestine of a patient with IBS. (**d**) The colonic TET tube in the intestine of a patient with UC. CD, Crohn’s disease; rCDI, recurrent *Clostridioides difficile* infection; IBS, irritable bowel syndrome; UC, ulcerative colitis; TET, transendoscopic enteral tubing.

Colonic TET was recommended by the most recent consensus from the FMT-standardization study group in Asia in 2019 [25] and an international FMT expert group in 2020 [26] due to its convenience and safety for WMT in clinical applications (Figure 2). A questionnaire analysis from Liang’s group indicated that an increasing proportion of the public were aware of FMT and had positive outlooks toward the use of FMT in the treatment of IBD and other diseases in recent years [27]. However, another questionnaire study about the recognition and attitudes of FMT through TET in patients with IBD revealed that a large proportion of participants were unaware of the concept of TET, suggesting that it is necessary to increase public attention and promote the medical application of colonic TET [28].

Generally, colonic TET is removed actively or falls out spontaneously after microbial therapy or medication treatment; the latter outcome is preferred in clinical practice. Due to the difference in the sample size in studies, the median retention time of colonic TET has been reported to be 12.4 days [12] and 8.6 days [19] in adults and 6 days in children [20]; the difference between adults and children can be attributed to the number and type of endoscopic clip. All of the existing studies indicate that endoscopic clips are an independent factor affecting the retention time. The reported success rate of performing colonic TET was 100% in both adult [19] and pediatric patients [20]. Moreover, physician–patient satisfaction [29] for colonic TET in adults was 97.8% [19], and the reasons for dissatisfaction were not mentioned in the relevant study. In children, the satisfaction rate was 100% [20]. 

Recently, Philip et al. reported on a patient with fulminant CDI requiring surgical loop ileostomy. The patient underwent rescue FMT, which was safely delivered by a Foley catheter through the ileostomy. The case highlighted the positive contribution of the Foley catheter in multiple FMTs, avoiding re-operation and unnecessary colonoscopy [30]. The role of the Foley catheter in this case was similar to colonic TET; however, it is not as stable as colonic TET because it is not fixed to the intestinal wall.

Several studies regarding FMT delivery via colonic TET demonstrated a high efficacy [31]. Zhou’s group indicated that in 47 patients with UC who underwent FMT treatment via colonic TET, the rate of steroid-free clinical response was 84.1% and steroid-free clinical remission was 70.5% at one month post-FMT [32]. Ding et al. demonstrated that the clinical response of UC patients one month post-FMT via colonic TET was 83.3% [3]. Moreover, Chen’s group reported that in 30 patients with active UC who underwent FMT via colonic TET and enema, the clinical response rate was 59.3% and the clinical remission rate was 40.7% [33]. Remarkably, there was no difference in efficacy between patients who underwent FMT via colonic TET or via other delivery routes (gastroscopy and nasojejunal TET) in different studies [3,34,35,36]. Nie’s group further demonstrated that regardless of whether it was gastroscopy or colonic TET, the delivery route might not affect fecal IgA-bacteria interactions after FMT [37].

Recently, it has been reported that two patients with IgA nephropathy who received FMT through colonic TET for 6–7 months both achieved partial clinical remission [38]. Furthermore, WMT via colonic TET was shown by He’s group to reduce the serum uric acid in patients with hyperuricemia and acute gout [39,40]. Details regarding the application of colonic TET in published articles are shown in Table 2. Of note, colonic TET is not recommended for traditional manual preparation of FMT because tube obstruction was reported while delivering the manual microbiota suspension in another study [41]. Recent reports have highlighted that WMT as a new methodology of FMT contributes to the decreased incidence of AEs compared with manual FMT [23]. Evidence suggests that colonic TET is an efficient, safe, and satisfying delivery route for FMT. 

### 3.2. Colonic TET for Drainage and Decompression from Deep Colon

Stricture formation and intestinal perforation are common complications of Crohn’s disease (CD) and results from the disease process, surgery, or drugs. The related practical recommendations about dealing with IBD-related strictures and perforation have been released by the International Interventional IBD Group [46,47]. Subsequently, the correct and prudent application of endoscopic stricturotomy and ileo-colonic resection have been emphasized by expert opinions [48,49]. Hence, the endoscopic procedure in the treatment of IBD-associated complications is still a challenge. Although endoscopic decompression of acute intestinal distension can reduce mortality in critically-ill patients [50], the trans-anal decompression tube should be applied discreetly as it may cause intestinal perforation [51]. Unlike traditional trans-anal depression tube placement in the left colon [52,53], colonic TET is a novel interventional procedure that will be promising for bringing benefits to patients under endoscopy and avoiding surgery.

Endoscopic perforations are usually observed in patients with CD-associated strictures [54]. The European Society of Gastrointestinal Endoscopy (ESGE) [55] and the International Intervention IBD Group [46] recommended that endoscopic intervention should be considered depending on the type and size of iatrogenic perforation. Moreover, diversion of the digestive luminal contents and decompression of tension pneumoperitoneum should be performed [55], and colonic TET could play a significant role in this situation. Endoscopy is the correct option if the perforation is found immediately; however, endoscopic reintervention can entail new risks and it may be difficult to locate the site of perforation. Furthermore, prompt diagnosis will affect patient outcomes, and if the endoscopic perforation is found too late, there is a risk of infection. Surgery must be performed once the patient shows symptoms of generalized peritonitis or sepsis. However, if a patient with acute colonic obstruction is in poor condition, emergency surgery compared with elective colon cancer resection has a higher mortality rate [56]. Therefore, placing a colonic TET as palliative treatment and awaiting a better time for surgery is necessary in critical illness.

Zhang et al. reported two cases of stricturing CD in the transverse colon in the same patient who underwent endoscopic balloon dilation and one case with UC and spreading mild dysplasia in the sigmoid colon, both patients suffered perforation after therapeutic endoscopy [17]. A colonic TET with loops was fixed to the ascending or descending colon wall with the intention of WMT and frequently delivering medications. However, perforation was identified by X-ray several days after endoscopy, and the colonic TET was immediately used for draining the air and fluid in the colon with syringe suction. Eventually, all patients recovered rapidly via colonic TET and were free from surgery [17]. In cases of intestinal pressure exceeding atmospheric pressure, regardless of whether surgery is necessary for the patient, colonic TET could be used to drain intestinal fluid and reduce the tension by opening the cap of the distal tube outside the intestine. In addition, colonic TET can be used to deliver antibiotics to prevent or treat infection. Of note, regular endoscopic procedures such as endoscopic mucosal resection within the colon and cap-assistant endoscopic sclerotherapy for hemorrhoids and prolapse have no effect on colonic TET [57].

## 4. Host-Microbiota Interaction Based on Sampling via Colonic TET

### 4.1. Discovery in Host–Microbiota Interaction

Microorganisms play critical roles in various physiological functions of the host. Exploring the human microbiota–host interaction could reflect the connection between health and disease [58,59]. Principally, all gut microbiota-derived metabolites are produced in one of three ways: directly from ingested compounds, from host-derived substrates, or de novo from primary metabolites [59]. Therefore, finding a more effective method to sample metabolites is vital. In most studies, fecal samples were used to study the gut microbiome. Although they can be acquired easily, continual fecal samples are rarely taken within short intervals [60]. Some studies found that the ileocecal microbiome, localized in the middle part of the gastrointestinal tract, had relatively higher diversity than the fecal microbiome [61]. Moreover, studies on sampling the intestinal lavage fluid (IVF) microbiome found that pathogenic microbiota was more abundant in the IVF than in feces, and the microbiome in the IVF may be a better indicator for evaluating the risk of developing colorectal cancer compared with fecal samples [62]. 

Microbial circadian rhythmicity is a feature of mammalian metabolism that might be a significant factor in the development of metabolic disease [63]. Lora V’s group found that the intestinal microbiota in the mouse small intestine programs diurnal metabolic rhythms through histone deacetylase 3 [64]. To investigate community dynamics in the intestine with better resolution, Wang et al. applied colonic TET to extract cecum fluid samples from healthy volunteers twice daily (10 a.m. and 10 p.m.) via syringe, from which metagenomic, metatranscriptomic, metabolomic, and virome analyses were conducted [65,66]. The results revealed the individuality of reconstruction in the microbiome composition, functions, and shared characteristics of the internal resilience of the gut microbiome. Sampling the ileocecal microbiota in situ provides unique insights into the diurnal patterns or circadian rhythms of the human gut microbiome for the first time. Based on samples from a healthy human cecum, Liu et al. further identified that gut microbial methionine impacts circadian clock gene expression and the reactive oxygen species level in the host gastrointestinal tract [18]. Moreover, Fawad et al. reported that gut microbe-generated short-chain fatty acids entrained intestinal epithelial circadian rhythms by inhibiting histone deacetylase [67]. Collection of intestinal fluid with a sterile syringe is recommended. Colonic TET is currently the best non-invasive tool for collecting microbial samples from the deep colon in humans.

### 4.2. Precision Delivery of Potential Microbiota and Its Metabolites

The interplay between the metabolic activities of the intestinal microbiome and its host forms a significant component of health [68]. The basis of this interaction is mediated by the release of microbially-derived metabolites that interact with the immune or metabolic systems of the host [69]. To date, accumulating evidence focusing on host microbiota has shown that some potential gut microbiota and derived metabolites can be used to reflect and treat different diseases [70]. For example, Meng’s group found that colitis could be treated with indole-3-propionic acid [70], which was also recently demonstrated by Serger et al. to promote nerve regeneration and repair in mice [71]. A 9-amino-acid peptide called D3 was designed by Zhao’s group, which could largely increase the abundance of Akkermansia muciniphila and downregulate CD36 to improve obesity [72]. Moreover, a study by Zhao’s group demonstrated that supplementation with Bacteroides uniformis improved autism spectrum disorder-like behaviors in a mouse model, which added new evidence for the host–microbiota interaction [73]. Table 3 presents some potential substances for the prevention or treatment of disease. In these studies, oral administration was the primary delivery. However, one possibility is that the concentration and properties of these substances had changed when they arrived at their destination in the intestine, which was not mentioned in the articles. Therefore, how to effectively transfer potential metabolites for treatment to the intestine has triggered further investigation.

To slow down the drug release in the acidic upper gastrointestinal tract after oral administration, novel prebiotic and postbiotic synergistic delivery microcapsules were proposed by Zhao’s team [82]. In addition, Zhong et al. proposed an orally deliverable strategy based on microalgae, which leveraged the biological properties of microalgal carriers to improve the bioavailability of loaded drugs for the treatment of colon cancer and colitis [85]. Similarly, Kaur et al. designed an orally administrable cargo transport device named bacterioboat, which consists of surface-encapsulated mesoporous nanoparticles on metabolically active *Lactobacillus reuteri* as a drug carrier that is suitable for oral administration [86]. In vivo studies have shown that the oral delivery of 5-fluorouracil via bacterioboat led to increased potency, resulting in improved shrinkage of solid tumors, enhanced life expectancy, and reduced side effects. Decorated bacteria for drug delivery in intestinal disease treatment and cancer therapy will be an innovative strategy in the future if we ignore the cost of development [87].

### 4.3. The Proof-of-Concept on Translational Microbial Research

A number of the exact mechanisms have not been verified in clinical research, and studies focusing on the interaction between the host or pharmacy and microbiota remain in the proof-of-concept stage [88]. Compared with the attempts of the precision delivery of potential metabolites and drugs, the advantage and feasibility of colonic TET as a novel delivery of the proof-of-concept in host–microbiota interactions and pharmacological research could be highlighted. Polyphenols mainly exist in plant-based food and are known to be beneficial in IBD alleviation. Singh et al. found that a combination of polyphenols from green tea extract and a prebiotic improved the beneficial gut microbiota (Lactobacillus, Bifidobacteria, Akkermansia, Roseburia spp.) abundance, restored Firmicutes/Bacteriodetes, and improved the Prevotella/Bacteroides proportions to effectively reduce the level of inflammation [89]. Enlightened by these studies, combined latent beneficial substances or FMT and probiotics via colonic TET might be a potential method to further explore the relationship between disease and gut microbiota [90]. 

## 5. Conclusions

Strategically choosing a new pathway like colonic TET might be more effective than traditional delivery methods in future research. Increasing studies have demonstrated that colonic TET is a promising, safe, and practical delivery method. The present review demonstrates the benefits of this novel technique in providing new options for the improvement of microbial therapy, rescue therapy for patients with endoscopic-related perforation, and research on dynamic host–microbiota interactions. There is no doubt that this review will improve the understanding of colonic TET for researchers, physicians, and patients in clinical practice and basic studies. 

## Figures and Tables

**Table 1:** Comparison of the different delivery methods.

Delivery Ways	Advantages	Limitations
Oral capsules	Overcome the concern of invasive administration; easy to perform	Efficacy may affect by gastric acid and the preservation state of bacteria
Gastroscopy	Easy to reach the target location	Not convenient to repeat FMTs
Mid-gut/Nasojejunal tube/PEGJ tube	Easy to reach the target location; convenient to repeat FMTs; easy to maintain	Placed under gastroscopy; limited and special population for use
Colonoscopy	Easy to reach the target location	Not convenient to repeat FMTs
Colonic TET tube	Easy to reach the target location; convenient to repeat FMTs; easy to maintain;	Placed under colonoscopy
Stoma in ilecolon/colon	Convenient to repeat FMTs; easy to perform	Only for selected population with surgical double-cavity stoma in ilecolon/colon
Enema	Easy to perform; low cost	Difficulty to hold the bacteria suspension in rectum for a long time

Abbreviations: PEGJ, percutaneous endoscopic gastrostomy with jejunal extension; FMT, fecal microbiota transplantation; TET, transendoscopic enteral tubing.

**Table 2:** The reported indications, clinical success rates, satisfaction rates, and adverse events of colonic TET.

Author, Year	Article Type	Case, *n*	Sex, Male, *n* (%); Age, Mean (Range), Years	Indication	Clinical Success Rate	Satisfaction Rate	The Mean Retention Time	Adverse Events	The Target Location	The Endoscopic Clips	The Average of Endoscopic Clips
Zhang et al., 2022 [32]	Prospective study	27	17(63.0%); 47.48 ± 12.34	UC	100%	NA	NA	NA	NA	NA	NA
Chen et al., 2021 [42]	Retrospective study	16	10 (62.5%); 39.88 ± 11	UC	100%	97.3%	NA	3	NA	NA	NA
Zhong et al., 2021 [20]	Prospective study	47	42 (89.36%); 5(4–6)	21 autism, 6 UC, 2 rCDI, 1 CD, 17 others	100%	100%	6 (5–7)	4	29 in ileocecal, 12 in the transverse colon, 6 in left colon ileum	35 in large, 12 in small	2 (1.75–3)
Long et al., 2020 [43]	Prospective study	257	138 (57%); 39.9 ± 18.4	132 UC, 14 CD, 10 epilepsy, 8 autism, 56 others	100%	NA	9.3 ± 3.8 (2–28)	21	215 in ileocecal, 6 in the transverse colon, 25 in the left colon, 6 in descending colon	154 in large, 103 in small	3.5 ± 1.0 (2–6) (in 95 cases)
Luo et al., 2020 [44]	Retrospective study	9	6 (66.7%); 47.44 ± 12.26	UC	100%	NA	NA	1	NA	NA	NA
Wen et al., 2020 [21]	Randomized controlled trial	303	155 (51.16%); 44.4 ± 17.6	93 constipation, 88 UC, 32 IBS, 9 CD, 2 health, 75 others	100%	100%	8 (6–10)	9	NA	NA	2.65 ± 1.1
Liu et al., 2021 [18]	Prospective study	5	NA	Health	100%	NA	NA	NA	5 in ileocecal	NA	NA
Chen et al., 2020 [33]	Prospective study	44	25 (57%); 44.4 ± 17.6	UC	100%	NA	NA	5	NA	NA	NA
Chen et al., 2020 [36]	Prospective study	5	5 (100%); 47.9 ± 10.6	UC	100%	NA	NA	0	NA	NA	NA
Zhang et al., 2019 [45]	Randomized controlled trial	21	NA; 49.2 ± 13.77	UC	100%	100%	NA	3	21 in ileocecal	NA	NA
Wang et al., 2019 [41]	Case series	5	4 (80%); 56.33 (31–94)	4 rCDI, 1 CD	100%	NA	NA	NA	5 in left colon	NA	NA
Zhang et al., 2021 [17]	Case	3	1 (50%); 38 (25–51)	2UC, 1 CD	100%	NA	NA	NA	1 in left colon, 2 in descending colon	NA	NA
Luo et al., 2021 [44]	Case	1	1 (100%); 32	UC	100%	NA	NA	NA	NA	NA	NA
Zhao et al., 2021 [38]	Case	2	0 (0%); 40 (32–48)	Refractory IgA nephropathy	100%	NA	NA	2	NA	NA	NA
Wang et al., 2020 [41]	Case	1	1 (100%); 77	rCDI	100%	NA	NA	NA	NA	NA	NA
Zhong et al., 2019 [20]	Case	1	1 (100%); 31	CD	100%	NA	NA	NA	NA	NA	NA
Zhang et al., 2019 [45]	Case	1	1 (100%); 55	UC	100%	NA	NA	NA	NA	NA	NA

Abbreviations: UC, ulcerative colitis; rCDI, recurrent Clostridioides difficile infection; CD, Crohn’s disease. NA, not applicable.

**Table 3:** The potential substances for the prevention or treatment of disease.

Item	Name	Model	Disease	Delivery Route	Possible Mechanism
Chemical substances and food	Acetylcholine	Mice and human	IBD	Enema	ACh promotes interleukin-10 secretion of monocytic myeloid-derived suppressor cells and suppresses the inflammation through activating the nAChR/ERK pathway [74]
	Polyphenol	Mice	Cancer	Oral administration	Oral administration of castalagin enriched for bacteria associated with efficient immunotherapeutic responses (Ruminococcaceae and Alistipes) and improved the CD8+/Foxp3+CD4+ ratio within the tumor microenvironment [75].
	Starch modified with acetate and butyrate	Human	Type 1 diabetes	Oral administration	Changes in gut microbiota composition, function, and immune profile following 6 weeks of starch supplementation were associated with increased SCFAs in stools and plasma [76].
Intestinal microbiota	Akkermansia	Mice	Aging	Oral administration	Oral administration of Akkermansia sufficiently ameliorated the senescence-related phenotype in the intestinal systems in aged mice and extended the health span [77]
	Saccharomyces Boulardii (Sb)	Mice	IBS	Gavage	Sb could upregulate SERT by EGFR activation and modulate gut microbiota to inhibit gut motility [78].
	Enterococcus	Mice	Cancer	Oral administration	Active enterococci express and secrete orthologs of the NlpC/p60 peptidoglycan hydrolase SagA that generate immune-active muropeptides. Expression of SagA in nonprotective E. faecalis was sufficient to promote anti-PD-1 antitumor efficacy [79].
	Eubacterium rectale	Mice	Lymphomagenesis	Oral administration	Producing butyrate to alleviate the TNF-induced TLR4/My88/NF-kB axis [80]
Metabolites	Acetate	Mice	Alzheimer’s disease	Oral administration	Acetate as the essential microbiome-derived SCFA driving microglia maturation and regulating the homeostatic metabolic state [81].
	9-amino-acid (D3)	Mice and macaques	Obesity	Oral administration	D3 ameliorated leptin resistance and upregulated the expression of uroguanylin (UGN), which suppresses appetite via the UGN-GUCY2C endocrine axis, and increased the abundance of Akkermansia [72].
	Indoles-3-propionic acid (IPA)	Human	Colitis	Oral administration	Modulating the gut microbiota, that is by significantly increasing the overall richness and abundance of short-chain fatty acids (SCFA) producing bacteria such as Faecalibacterium and Roseburia [82].
Phage	Kp2-phage	Mice and human	Intestinal inflammation	Oral administration	Proof-of-concept assessment of Kp-targeting phages in an artificial human gut and in healthy volunteers demonstrates gastric acid-dependent phage resilience, safety, and viability in the lower gut [83].
	Enterococcal bacteriophage	Mice	Tumor	Oral administration	In mouse models, administration of enterococci containing the bacteriophage boosted T cell responses after treatment with chemotherapy or programmed cell death protein 1 (PD-1) blockade. In humans, the presence of the bacteriophage was associated with improved survival after PD-1 immunotherapy [84].

Abbreviations: IBD, inflammatory bowel disease; IBS, irritable bowel syndrome. SCFA, short-chain fatty acid; PD-1, programmed cell death protein 1; UGN, uroguanylin.

## Data Availability

Not applicable.
